# Gallstone Decalcification and Dissolution Using Chenodeoxycholate and Citrate

**DOI:** 10.1155/1990/96130

**Published:** 1990

**Authors:** Avni Sali, Luis Vitetta

**Affiliations:** 1 Department of Surgery The University of Melbourne Repatriation General Hospital Heidelberg Victoria 3081 Australia; 2 University Department of Surgery Repatriation General Hospital Heidelberg Victoria 3081 Australia

## Abstract

Gallstone dissolution may be possible only in selected patients. Patients with calcified or large gallstones are not suitable for dissolution. Citrate is normally present in bile and an oral citrate load can increase biliary citrate.

A combination of chenodeoxycholic acid (C.D.C.A.) and citrate has been shown to dissolve calcified cholesterol gallstones *in vitro*. Patients with calcified or large gallstones were treated with a combination of C.D.C.A. and citrate. Partial decalcification was achieved in seven out of twenty patients with calcified stones (35%) and complete decalcification in four patients (20%). One of the patients with large stones had complete dissolution.

Five patients who were suitable for C.D.C.A. treatment but did not respond were also treated with C.D.C.A. and citrate. One of the patients in this latter group had complete dissolution. Oral citrate can decalcify some calcified gallstones.


*HPB Surgery*, 1990, Vol. 3, pp. 59–65
Reprints available directly from the publisher
Photocopying permitted by license only

© 1990 Harwood Academic Publishers GmbH
Printed in the United Kingdom

### GALLSTONE DECALCIFICATION AND DISSOLUTION USING CHENODEOXYCHOLATE AND CITRATE

AVNI SALI* and LUIS VITETTA

*Department of Surgery, The University of Melbourne, Repatriation General Hospital, Heidelberg, Victoria, 3081, Australia.*

*(Received 29 March 1990)*

Gallstone dissolution may be possible only in selected patients. Patients with calcified or large gallstones are not suitable for dissolution. Citrate is normally present in bile and an oral citrate load can increase biliary citrate.

A combination of chenodeoxycholic acid (C.D.C.A.) and citrate has been shown to dissolve calcified cholesterol gallstones *in vitro*. Patients with calcified or large gallstones were treated with a combination of C.D.C.A. and citrate. Partial decalcification was achieved in seven out of twenty patients with calcified stones (35%) and complete decalcification in four patients (20%). One of the patients with large stones had complete dissolution.

Five patients who were suitable for C.D.C.A. treatment but did not respond were also treated with C.D.C.A. and citrate. One of the patients in this latter group had complete dissolution. Oral citrate can decalcify some calcified gallstones.

KEY WORDS: Gallstone dissolution, decalcification, chenodeoxycholate, citrate

### INTRODUCTION

It is now well established that, if certain criteria are adhered to, patients with radio lucent gall bladder stones can have their stones dissolved with oral bile acid therapy. However, up to the present time it has not been possible to dissolve calcified gallstones using oral treatment. The potential of gallstone lithotripsy in the treatment of gall bladder stones and the subsequent need for dissolution of the resulting gallstone fragments has increased interest in gallstone dissolution.

It was first shown in 1980 by Sali and co-workers that citrate is normally present in bile and also that citrate concentration of bile can be increased by an oral citrate load[1]. Subsequent *in vitro* studies by this group have shown that calcified pigment stones can be dissolved using citrate solutions alone and calcified cholesterol gallstones can be dissolved using solutions containing both citrate and bile acid[2,3].

The above findings stimulated a therapeutic trial to dissolve calcified gall bladder gallstones using oral citrate and chenodeoxycholic acid (C.D.C.A.). A second group of patients with large gallstones generally considered not suitable for treatment with bile acid therapy plus a smaller group who were resistant to bile acid therapy were also studied.

*Address for correspondence: Mr. A. Sali, University Department of Surgery, Repatriation General Hospital, Heidelberg, Victoria, 3081, Australia.

### METHODS AND RESULTS

Three groups of patients were treated for at least 6 months.

Group 1: 20 patients with calcified gall bladder stones (Table 1).

**Table 1** Patients with calcified stones (Group 1)

| *Patient No.* | *Sex* | *Age (years)* | *Calcified stones (No.)* | *Diameter largest stone (cm)* | *Calcification* | | | *Decalcification* | | | *Duration of Treatment (months)* |
|---|---|---|---|---|---|---|---|---|---|---|---|
| | | | | | *Central* | *Peripheral* | *Diffuse* | *Partial* | *Complete* | *No Change* | |
| 1 | F | 64 | 3 | 1.8 | + | | | | | + | 12 |
| 2 | F | 52 | 9 | 2 | | | + | | | + | 6 |
| 3 | F | 47 | 10 | 0.3 | | | + | | | + | 8 |
| 4 | F | 56 | 1 | 2.5 | | + | | + | | | 9 |
| 5 | F | 29 | 2 | 1 | + | | | + | | | 12 |
| 6 | F | 69 | 1 | 1.2 | | | + | + | | | 7 |
| 7 | M | 63 | >20 | 0.6 | + | | | + | | | 12 |
| 8 | M | 75 | 1 | 1.2 | | + | | + | | | 12 |
| 9 | M | 65 | >20 | 0.5 | + | | | + | | | 8 |
| 10 | F | 66 | 3 | 2.2 | | + | | | +* | | 24 |
| 11 | F | 32 | 7 | 1 | | + | | | | + | 9 |
| 12 | F | 32 | >20 | 1.5 | | + | | | | + | 12 |
| 13 | F | 57 | 4 | 1.2 | | + | | | | + | 12 |
| 14 | F | 33 | 2 | 0.7 | | | + | | + | | 18 |
| 15 | F | 69 | 2 | 1.4 | | + | | | + | | 8 |
| 16 | F | 81 | >20 | 0.2 | | | + | | | + | 7 |
| 17 | ŒF | 55 | 1 | 2 | | + | | + | | | 12 |
| 18 | F | 37 | 1 | 1.5 | | + | | | + | | 18 |
| 19 | F | 39 | 11 | 1.5 | | + | | | | + | 18 |
| 20 | F | 48 | 1 | 2.5 | | + | | | | + | 6 |

* Did not have follow-up ultrasound.

Group 2: 12 patients with stones greater than the 2 cm. (Table 2).

**Table 2** Patients with large radiolucent gallstones (Group 2)

| *Patient No.* | *Sex* | *Age (years)* | *Calcified stones (No.)* | *Diameter largest stone (cm)* | *Dissolution* | | | *Duration of Treatment (months)* |
|---|---|---|---|---|---|---|---|---|
| | | | | | *Partial* | *Complete* | *No Change* | |
| 1 | M | 33 | 2 | 2 | | + | | 24 |
| 2 | M | 51 | 2 | 2.2 | | | + | 12 |
| 3 | F | 45 | 1 | 2.2 | | | + | 12 |
| 4 | M | 41 | 1 | 2.3 | | | + | 12 |
| 5 | M | 38 | 5 | 3.0 | | | + | 8 |
| 6 | F | 54 | 1 | 2.2 | + | | | 12 |
| 7 | F | 41 | >20 | 2.2 | | | + | 12 |
| 8 | F | 50 | 2 | 2 | | | + | 12 |
| 9 | M | 52 | 1 | 2.3 | | | + | 9 |
| 10 | F | 23 | 2 | 2.3 | + | | | 18 |
| 11 | F | 29 | 1 | 2 | | | + | 12 |
| 12 | M | 75 | 2 | 2 | | | + | 12 |

Group 3: 5 patients with stones less than 1.5 c.m. that had not responded to C.D.C.A. treatment. (Table 3).

**Table 3** Patients not responding to CDCA (Group 3) then treated with Citrate and CDCA

| *Patient No.* | *Sex* | *Age (years)* | *No. of stone (cm)* | *Diameter largest stone CDCA* | *Treatment (months)* *citrate* | *CDCA+ Partial* | *Dissolution* *Complete* | *No change* |
|---|---|---|---|---|---|---|---|---|
| 1 | F | 40 | 1 | 0.8 | 6 | 9 | | + |
| 2 | F | 37 | 5 | 0.4 | 6 | 6 | | + |
| 3 | M | 59 | 1 | 0.5 | 6 | 6 | | + |
| 4 | F | 63 | 8 | 1.5 | 6 | 6 | | + |
| 5 | F | 62 | 1 | 1.5 | 6 | 12 | + | |

Both Group One and Two are generally regarded as unsuitable for treatment with bile acid therapy alone.

The Group 3 patients were initially treated with C.D.C.A. alone and after no response at the end of 6 months they were treated with a combination of C.D.C.A. and citrate. For all groups of patients the dose of C.D.C.A. was 14–16 mg per kilogram given in two doses daily, whereas the dose of citrate was 90 mmol per day, also given in two doses. A number of patients withdrew from the study before three months and they have not been included in the treatment group. From Group 1, 7 patients ceased treatment because of diarrhoea, and another two patients stopped treatment as one required a cholecystectomy and the other had to have surgery for an aortic aneurysm. Diarrhoea is a well recognized problem in up to 40% of patients having C.D.C.A. Ursodeoxycholate (U.C.D.A.) has not been available in Australia. In Group 2, one patient ceased treatment because of diarrhoea and another two were unable to tolerate the citrate taste which was given to the patients in liquid form. Another patient decided to have a cholecystectomy after being on dissolution treatment for five months. No patients withdrew from the third group. Clinical details of the patients remaining in the three groups are shown in Tables 1–3.

All patients had an oral cholecystogram as well as an ultrasound before starting treatment to ensure that they had a functioning gall bladder. Blood was taken before treatment was started and again six weeks later for liver function tests (serum bilirubin, alanine transaminase, gamma — glutamyl transpeptidase and alkaline phosphatase), haemoglobin, white blood and platelet counts, and electrolytes (sodium, potassium, bicarbonate, urea, creatinine, calcium, phosphate).

The effect of the combination treatment of C.D.C.A. and citrate on gallstone dissolution, decalcification or disappearance was assessed by a follow-up oral cholecystogram as well as ultrasonography after six months treatment. Treatment was generally well tolerated. Of the patients in Group 1, partial decalcification in seven of the twenty patients (35%) was achieved. Complete decalcification occurred in four (20%) of 20 patients and two of these patients had no stones on oral cholecystography. Of these latter two patients, one had smaller stones on ultrasonography but the other patient, who was from a distant town, refused to have an ultrasound because of the necessary travel. Complete dissolution occurred in one patient in Group 2 and partial dissolution occurred in two patients. One of the five patients in Group 3 had complete dissolution. No haematological abnormalities or persistent bio-chemical evidence of hepatotoxicity was noted in any of the patients.

### DISCUSSION

Although this was not a controlled study it showed an interesting response to treatment with a combination of C.D.C.A. and citrate in patients with calcified stones. Complete decalcification occurred in four (20%) and partial decalcification in seven (35%). Radio opaque gallstones do not dissolve with oral bile acid therapy whether calcification is present in the periphery, centre or throughout the stones. A few gallstones with a tiny calcified nucleus of less than 3 mm. in diameter have been reported to have been dissolved with bile acid therapy. It is very likely that these small stones passed into the duodenum without the calcified centre being dissolved. Calcified rims of gallstones can be partially decalcified with Rowachol, which is an essential oil preparation[5].

Oral citrate is excreted in pancreatic juice and citrate has been shown to dissolve calcified pancreatic stones both *in vitro* and in humans[6,7]. The mode of action of citrate in the dissolution of pancreatic stones may be similar to that which decalcifies calcified gall bladder stones. Citrate is known to form soluble complexes with calcium and therefore calcified stones are probably decalcified because of this characteristic.

Four patients had complete decalcification of their stones as judged by X-ray. However, three of these patients had stones still present on ultrasonography. It is possible that the patient with small diffusely calcified stones had calcified pigment stones with a lattice resisting dissolution remaining after calcium and, perhaps, cholesterol removal.

It was disappointing that there was a poor response with citrate and C.D.C.A. therapy in those patients with stones too large to be suitable for bile acid treatment alone. There was also a poor response in those with smaller radiolucent stones where there had been no response to C.D.C.A. therapy alone. Only one of the patients with large radiolucent stones had complete dissolution and another partial dissolution. One of the five patients with stones resistant to C.D.C.A. therapy had complete dissolution of her stone when citrate was added to the treatment. It is possible that three of the non-responders in this latter group of patients may have had black pigment stones as their stones were less than 8 mm in diameter, although only one of these patients had multiple stones. The other non-responder had eight stones, all of which were approximately 1.5 cm in diameter. It is therefore likely that more than six months therapy with C.D.C.A. and citrate was necessary to achieve a response in this latter patient.

The majority of radiolucent stones that do not respond to C.D.C.A. therapy have significant calcium compound on the stone surface which would prevent cholesterol dissolution[8]. It had been hoped that a better response rate would have been achieved as we were able to dissolve calcified cholesterol stones *in vitro* using bile acid and citrate but not by using bile acid alone[2,3]. Calcium bilirubinate is the principle pigment of pigment gallstones and an amorphous matrix of calcium bilirubinate and protein has been found at the centre of most cholesterol gallstones[9,10]. It is likely that this central nucleus of calcium bilirubinate is important for cholesterol precipitation and stone formation. Therefore the concentration of citrate which is normally present in bile may be an important factor in maintaining calcium solubility and hence the prevention of gallstone formation. Oral citrate has been used to prevent the formation of calcified kidney stones and it may be possible to use citrate for the prevention of gallstone recurrence following dissolution[11].

Citrate and C.D.C.A. may also be useful in dissolving calcified gallstone fragments following lithotripsy.

*References*

1. Sali, A., Crowe, C., Iser, J. Donohue, W. and Kune, G. (1980) Biliary citrate secretion. *Australian and New Zealand Journal of Medicine*, **10**, 114
2. Donohue, W., Hocking, C., Lawrance, S. Iser, J. and Sali, A. (1981) Citrate and *in vitro* gallstone dissolution. *Australian and New Zealand Journal of Surgery*. **51**, 396
3. Vitetta, L. (1984) Gallstone composition and dissolution. Ph.D. Thesis. Volume 2, Chapter 6
4. Schoenfield, L.J. and Lachin, J.N. (1981) The Steering Committee and National Co-Op Gallstone Study Group. Chenodiol (chenodeoxycholic acid) for dissolution of gallstones: The National Co-Op Gallstone Study. *Annals Intern Medicine*, **95**, 257–282
5. Ellis, W.R., Rose, D.H., and Richmond, C.R. *et al.* (1980) Radio opaque gallstones — reduction in size and calcium content on treatment with Rowachol. *Gut*, **21**, A910
6. Lohse, J. Verine, H.J. and Sarles, H. (1981) Studies on pancreatic stones. *In-vitro* dissolution. *Digestion*, **21**, 125–132
7. Sarles, H. Verine, H.J. Hohse, J., Sahel, J., De Garo, A. and Zerolo, J. (1979) *In vivo* dissolution of pancreatic stones through prolonged citrate administration. *Gastroenterology*, **76**, 1235
8. Whiting, M.J., Jarvinin, V. and Watts, J.McK. (1980) Chemical composition of gallstones resistant to dissolution therapy with chenodeoxycholic acid. *Gut*, **21**, 1077–1081
9. Bogren, H. and Larsson, K. (1963) On the pigment in biliary calculi. *Scandinavian Journal of Clinical and Laboratory Investigation*, **15**, 569–572
10. Been, J.M., Bills, P.M. and Lewis, D. (1979) Microstructure of gallstones. *Gastroenterology*, **76**, 548–555
11. Marwick, C. (1983) New drugs selectively inhibit kidney stone formation. *Journal of American Medical Association*, **250**, 321–322

*(Accepted by Miles Little 22 March 1990)*

### INVITED COMMENTARY

The fact that gallstones are not homogeneous in their chemical composition poses a significant problem for gallstone therapy. The presence of calcium salts, mainly carbonate and/or bilirubinate, in cholesterol gallstones has been recognized as a strong negative factor for dissolution treatment with bile acids[1]. Precipitated calcium salts may be present in the centre of stones, after acting as nucleating factors to initiate gallstone growth, or calcification may occur on the surface or rim of cholesterol gallstones after a period of stone growth. Bile acid treatment with ursodeoxycholic acid, but not chenodeoxycholic acid, may actually promote stone calcification, which has been observed in up to 12% of patients taking ursodeoxycholic acid orally[2,3].

In an effort to overcome the inhibitory effect of calcification on stone dissolution, several potential stone solvents have been investigated in laboratory studies[4,5]. In particular, the calcium-chelating agent ethylenediamine-tetra acetic acid (EDTA) has been examined as an alternating treatment with bile acids, but bile acid-EDTA

solutions were not found to be effective on calcified cholesterol stones[4]. Although pigment stones are disaggregated by bile acid-EDTA treatment *in vitro*[4], no *in vivo* studies have been performed to assess the efficacy of this combination for gallstone dissolution. The work of Sali and Vitetta is therefore of interest because citrate may act as a calcium-chelating agent in a similar fashion to EDTA, and it has been previously shown that an oral citrate load can increase the citrate concentration in bile.

The most important finding of the work of Sali and Vitetta is that a high incidence of stone decalcification was observed after combined treatment with oral chenodeoxycholic acid and citrate for at least six months. Unfortunately this was not associated with a noticeable increase in the incidence of gallstone dissolution over the low incidence which was expected from chenodeoxycholic acid treatment alone. This finding is somewhat surprising and suggests that either calcification was not the main factor preventing gallstone dissolution in many patients or that longer treatment may have been required to effect dissolution. Thus, it has not been shown that oral citrate is of any benefit in promoting gallstone dissolution with chenodeoxycholic acid, but it is theoretically possible that it could be useful in preventing gallstone recurrence in successfully treated patients, since calcium solubility in bile is likely to be of critical importance in the formation of gallstones[6].

Malcolm J. Whiting, PhD., M.A.A.C.B.,
Department of Biochemistry and Chemical Pathology,
Flinders Medical Centre,
Bedford Park, South Australia, 5042.

*References*

1. Schoenfield, L.J., Lachin, J. (1981) The Steering Committee, and the National Co-Operative Gallstone Study Group: Chenodiol (Chenodeoxycholic acid) for dissolution of gallstones: The National Co-Operative Gallstone Study. *Ann. Int. Med.* **95**, 257–82
2. Podda, M., Zuin, M., Battezzati, P.M. *et al.* (1989) Efficacy and safety of a combination of chenodeoxycholic acid and ursodeoxycholic acid for gallstone dissolution: a comparison with ursodeoxycholic acid alone. *Gastroenterology*, **96**, 222–9
3. Bateson, M.C., Bouchier, I.A.D., Trash, D.B. *et al.* (1981) Calcification of radiolucent gallstones during treatment with ursodeoxycholic acid. *Brit. Med. J.*, **283**, 645–6
4. Leuschner, U., Wosiewitz, U., Baumgartel, H., *et al.* (1988) Dissolution of calcified cholesterol stones and of brown and black pigment stones of the gallbladder. *Digestion*, **39**, 100–10
5. Wosiewitz, U., Gulduтuna, S., Fischer, H. *et al.* (1989) Pigment gallstone dissolution *in vitro*. *Scand. J. Gastro.* **24**, 373–80
6. Rege, R.V., Moore, E.W. (1986) Pathogenesis of calcium-containing gallstones. *J. Clin. Invest.* **77**, 21–6

### INVITED COMMENTARY

Gallstone dissolution is appropriate for fewer than one in three patients with gallstones[1]. Complete dissolution of calculi will subsequently occur in only 50% of the patients treated. Even if the stones are successfully dissolved life table analysis shows a cumulative recurrence rate of 10% a year over the first 4 years[2]. Possible reasons for incomplete dissolution include gallstone size and composition, patient compliance, and inability to unsaturate cholesterol from the bile.

The authors have addressed two groups of patients not usually amenable to dissolution therapy — those with calcified and large calculi. Encouraged by earlier *in vitro* studies where they were able to decalcify stones containing up to 20% calcium, Sali and Vitetta have added an oral citrate load to chenodeoxycholic acid (CDCA). Thirty seven patients completed the treatment protocol, whilst 9 withdrew from Group 1 and 4 from Group 2. An optimal dose of CDCA was used[1]. The time period chosen for the study allowed sufficient time for large stones to dissolve. The most important findings were from Group 1 where 4 patients showed complete decalcification of their gallstones and the effect was not restricted to small stones. Whether the effect of citrate is direct or whether it acts as an adjunct to CDCA, as terpenes do, is not clear. Treatment of patients with large stones with this combination of CDCA and citrate was less promising. Only 3 of the 12 patients responded despite a median of 12 months treatment.

The authors have demonstrated a means of de-calcifying and subsequently dissolving some calcified gallstones. Whilst further studies will be needed to confirm these preliminary findings, the ability to dissolve calcified stones has been demonstrated *in vivo* for what appears to be the first time. In the meantime, cholecystectomy remains the gold standard against which non-operative techniques must be judged.

Michael Hollands
Department of Surgery
Westmead Centre
WESTMEAD, NSW 2145
Australia

*References*

1. Fromm, H. (1986) *Gastroenterol*, **91**, 1560–7
2. O'Donnell, L.D.J. and Heaton, K.W. (1988) *Gut*, **29**, 655–8

